# Shutdown of ER-associated degradation pathway rescues functions of mutant iduronate 2-sulfatase linked to mucopolysaccharidosis type II

**DOI:** 10.1038/s41419-018-0871-8

**Published:** 2018-07-24

**Authors:** Yosuke Osaki, Atsushi Saito, Soshi Kanemoto, Masayuki Kaneko, Koji Matsuhisa, Rie Asada, Takao Masaki, Kenji Orii, Toshiyuki Fukao, Shunji Tomatsu, Kazunori Imaizumi

**Affiliations:** 1 Department of Biochemistry, Institute of Biomedical & Health Sciences, Hiroshima University, 1-2-3 Kasumi, Minami-ku, Hiroshima 734-8553 Japan; 2 Department of Stress Protein Processing, Institute of Biomedical & Health Sciences, Hiroshima University, 1-2-3 Kasumi, Minami-ku, Hiroshima 734-8553 Japan; 3 Department of Nephrology, Hiroshima University Hospital, 1-2-3 Kasumi, Minami-ku, Hiroshima 734-8553 Japan; 4 Department of Pediatrics, Graduate School of Medicine, Gifu University, 1-1 Yanagido, Gifu, 501-1193 Japan; 5 Department of Biological Sciences, University of Delaware, 118 Wolf Hall, Newark, Delaware 19716 USA; 6 Nemours/Alfred I. duPont Hospital for Children, 1600 Rockland Road, Wilmington, Delaware 19803 USA

## Abstract

Mucopolysaccharidosis type II (MPS II), also known as Hunter syndrome, is a devastating progressive disease caused by mutations in the iduronate 2-sulfatase (*IDS*) gene. IDS is one of the sulfatase enzymes required for lysosomal degradation of glycosaminoglycans. Mutant proteins linked to diseases are often prone to misfolding. These misfolded proteins accumulate in the endoplasmic reticulum (ER) and are degraded by the ubiquitin–proteasome pathway (ER-associated degradation (ERAD)). The decreased enzyme activities of IDS mutants may be due to accelerated degradation by ERAD. However, intracellular dynamics including degradation of IDS mutants is unexplored. In this report, we examined biochemical and biological characteristics of wild-type (WT) IDS and IDS mutants expressed in HeLa cells. IDS was shown to be glycosylated in the ER and Golgi apparatus and proteolytically cleaved to generate the mature forms in the Golgi apparatus. The mature WT IDS was translocated to the lysosome. In contrast, all IDS mutants we examined were found to accumulate in the ER and could not efficiently translocate to the lysosome. Accumulated IDS mutants in the ER were ubiquitinated by ERAD-related ubiquitin E3 ligase HRD1 followed by degradation via ERAD. Suppressed degradation of ‘attenuated’ mutant A85T IDS (the late-onset form of MPS II) by inhibiting ERAD components improved translocation to the lysosome and its activities. Our novel findings provide alternative targets to current principal therapies for MPS II. These perspectives provide a potenti al framework to develop fundamental therapeutic strategies and agents.

## Introduction

Mucopolysaccharidoses (MPSs) are a group of lysosomal storage disorders (LSDs) caused by the absence or malfunction of lysosomal enzymes^1^. One of the major MPSs is mucopolysaccharidosis type II (MPS II) (also known as Hunter syndrome)^2^. MPS II is an X-linked genetic disorder caused by a deficiency of iduronate 2-sulfatase (IDS)^3,4^. IDS is a sulfatase enzyme located in the lysosome and is required for lysosomal degradation of heparan sulfate and dermatan sulfate of glycosaminoglycans (GAGs). MPS II is characterized by the progressive lysosomal accumulation of GAGs because of the loss of IDS function. Patients with MPS II exhibit systemic manifestations including skeletal deformities, mental retardation, rigid joints, a thick skin, hepatosplenomegaly and valvular heart disease. In general, the progression of the disease shows two major clinical phenotypes. The ‘severe’ early-onset form appears within 2–4 years of age. Patients exhibit behavioral disturbances and die before reaching adulthood. In contrast, patients with the ‘attenuated’ late-onset form exhibit latent or slowly impaired systemic manifestations, including skeletal deformities and heart disease and survive into late adulthood^4^. Enzyme replacement therapy with recombinant human IDS and hematopoietic stem cell transplantation are the current standard treatments for MPS II patients^5–8^. These therapeutic strategies partially improve some clinical symptoms such as hepatosplenomegaly and joint mobility. However, they do not alleviate other major symptoms including skeletal deformities and mental retardation^9–11^. Thus, current therapies lack efficacy for all symptoms and new therapeutic strategies with feasibility are required.

The *IDS* gene encodes a protein that is 550 amino acids in length that shows high homology with the sulfatase protein family^12,13^. The 75 kDa precursor forms synthesized in the endoplasmic reticulum (ER) are processed to yield the 55 kDa mature forms, followed by relocation into the lysosome^14–16^. Currently, over 500 mutations of the *IDS* gene have been reported^17,18^. Mutants that display the attenuated phenotype of MPS II show residual enzyme activities (0.2–2.4% of the wild-type (WT) IDS activities), whereas those mutants exhibiting the severe phenotype have little or no activities^19^. Many of these mutants potentially cause misfolding of the encoded IDS protein. Misfolded proteins are known to accumulate in the ER and are degraded by the ubiquitin–proteasome pathway, known as ER-associated degradation (ERAD)^20–22^. IDS mutants are possibly degraded by the ERAD system, which is accompanied by a reduction in intracellular enzyme activities of IDS.

ERAD degrades unfolded proteins that accumulate in the ER lumen^20^. Unfolded proteins in the ER lumen are exported into the cytosol via retrotranslocation. During retrotranslocation, unfolded proteins are conjugated with ubiquitin chains by ER-associated ubiquitin E3 ligases on the cytosolic side of the ER membrane. Ubiquitinated unfolded proteins are extracted from the membrane and delivered to the 26S proteasome for degradation^20–22^. Mutant proteins linked to several diseases are degraded by ERAD. Those mutants include cystic fibrosis transmembrane conductance regulator (CFTR) (cystic fibrosis)^23–26^, aquaporin-2 (nephrogenic diabetes insipidus)^27^, HMG-CoA reductase (atherosclerosis)^28^ and a lysosomal enzyme β-glucocerebrosidase (GCase) (Gaucher’s disease)^29,30^. Therefore, understanding detailed mechanisms for regulating the degradation of mutant proteins via ERAD are crucial for developing therapeutic strategies that target those diseases.

In this study, we have analyzed biochemical characteristics and intracellular dynamics of WT IDS and IDS mutants. All IDS mutants were retained in the ER and rapidly degraded by ERAD, and translocation to the lysosome was barely observed. Shutdown of the ERAD pathway using small interfering RNAs (siRNAs) against ERAD components partially recovered both translocation of the IDS mutants to the lysosome and the enzyme activities.

## Results

### Disturbed proteolytic cleavage of IDS mutants

**Fig. 1:**
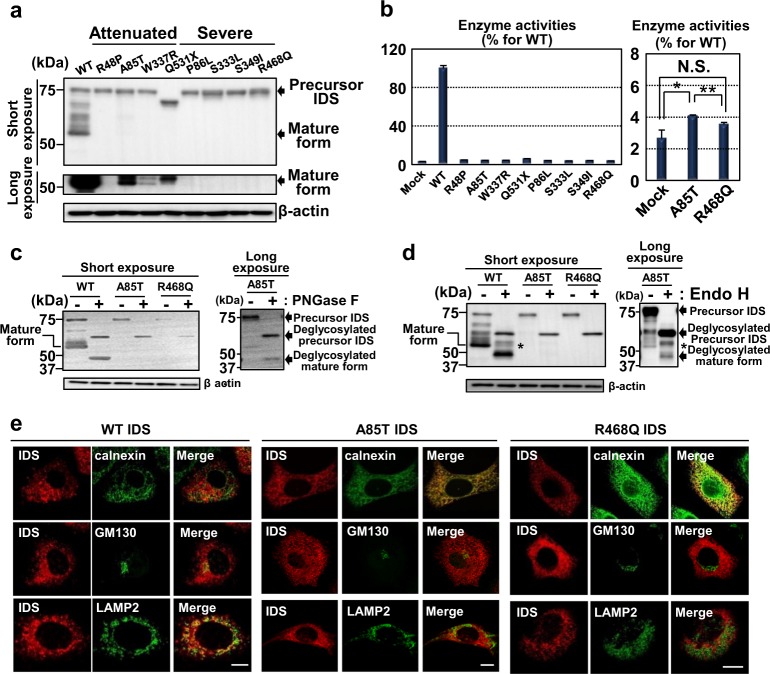
IDS mutants do not receive proteolytic cleavage and accumulate in the ER. **a** Western blotting analysis of IDS in HeLa cells expressing WT IDS or IDS mutants (*N* = 3). The 75 kDa precursor forms and the 55 kDa mature forms appeared in cells expressing WT IDS. The levels of the mature forms of attenuated mutants (R48P, A85T, W337R and Q531X) were barely detected by long exposure times. The mature forms of severe mutants (P86L, S333L, S349I and R468Q) could not be observed. **b** Enzyme activities of endogenous IDS in HeLa cells and exogenously expressed WT IDS or IDS mutants. The enzyme activities of both attenuated and severe IDS mutants were low, compared with that of WT IDS. The enzyme activities of the attenuated A85T IDS mutant were slightly higher than that of the severe R468Q IDS mutant. Mock indicates empty vector (mean ± SD, *N* = 3, Student’s *t*-test, *P < 0.05, **P < 0.01). **c**, **d** Western blotting analysis of IDS in HeLa cells expressing WT IDS or IDS mutants. Cell lysates were treated with **c** PNGase F for 1 h (*N* = 3) and (**d**) Endo H for 1 h (*N* = 3). The asterisk indicates Endo H-resistant mature forms of IDS. **e** Immunofluorescence staining analysis of IDS, calnexin, GM130 and LAMP2 in HeLa cells expressing WT IDS or IDS mutants. Bars: 10 µm

We carried out western blotting analysis using lysates of HeLa cells transfected with WT IDS or IDS mutants (R48P, A85T, W337R, Q531X, P86L, S333L, S349I and R468Q) (Fig. 1a). The 75 kDa precursor forms and the 55 kDa mature forms appeared in cells expressing WT IDS. Although the expression levels of the precursor forms were comparable with those of the WT IDS, the amounts of the mature forms of the ‘attenuated’ mutants (R48P, A85T, W337R and Q531X) were barely detected by short exposure times but were clearly detected by longer exposure times. These data indicate that small amounts of several attenuated mutants are cleaved to become mature forms. The efficiency of the cleavage differs among the attenuated mutants. In contrast to the attenuated mutants, the mature forms of all ‘severe’ mutants (P86L, S333L, S349I and R468Q) were not observed.

It has been reported that defective processing of IDS mutants results in the absence of enzyme activities of IDS in CHO cells^19^. Endogenous IDS in HeLa cells showed a very low level of enzyme activities, compared with those in HeLa cells expressing exogenous WT IDS (Fig. 1b, left panel). A85T (one of the attenuated mutants) IDS exhibited a high level of the enzyme activities, compared with that of endogenous IDS in HeLa cells (Fig. 1b, right panel). The enzyme activities of A85T IDS were slightly, but significantly, higher than that of the severe mutant R468Q IDS (little or no enzyme activities). The level of enzyme activities for each IDS we examined was comparatively consistent with the amount of the mature forms. Therefore, proteolytic cleavage is crucial for regulating the enzyme activities of IDS. In the following experiments, we mainly focused on A85T (attenuated mutant) and R468Q (severe mutant) IDS mutants because these two mutations are most commonly found in MPS II patients in various regions including Europe and Asia^31–34^.

### IDS is glycosylated at the ER and Golgi apparatus

We next examined the glycosylation of IDS. The precursor 75 kDa WT IDS and IDS mutants were shifted to approximately 60 kDa by treatment with PNGase F, which cleaves all N-linked carbohydrates (Fig. 1c). The 55 kDa mature forms of WT and A85T IDS were also shifted to approximately 40 kDa by glycolytic treatment (see the long exposure column for the mature forms of A85T IDS). The mature forms and the deglycosylated forms of R468Q IDS were not observed, although the precursor forms had N-linked glycosylation. Treatment with Endo H, which cleaves high mannose types of N-linked carbohydrates but does not cleave complex types of N-linked carbohydrates modified in the trans-Golgi apparatus, caused all types of precursors (WT, A85T and R468Q) and mature forms of WT IDS and A85T IDS to have lower molecular weights (see the long exposure column for mature forms of A85T IDS) (Fig. 1d). Partial carbohydrates conjugated to mature forms of WT IDS and A85T IDS were resistant to cleavage by Endo H, indicating that these mature forms may be modified by complex types of carbohydrates at the trans-Golgi apparatus.

### IDS mutants are prone to localize in the ER but not in lysosomes

The immunoreactivities of almost all WT IDS were observed as lysosome-associated membrane protein 2 (LAMP2; lysosomal marker)-positive dot structures (Fig. 1e). Partial immunoreactivities also coincided with those of GM130 (*cis-*Golgi apparatus marker). In contrast, the immunoreactivities of calnexin (ER marker) barely overlapped with that of WT IDS. A85T IDS was slightly detected in the LAMP2-positive lysosome and GM130-positive *cis-*Golgi apparatus, whereas the immunoreactivities overlapped with calnexin were markedly higher, compared with that of WT IDS. R468Q IDS showed a significant decrease in localization to both the *cis-*Golgi apparatus and the lysosome. These findings suggest that A85T IDS and R468Q IDS are prone to remain in the ER. Accumulation of unfolded proteins including misfolded mutant proteins inside the ER leads to ER stress^35,36^. The three major canonical branches of the unfolded protein response (UPR) are protein kinase R-like ER kinase (PERK), inositol-requiring kinase 1 (IRE1) and activating transcription factor 6 (ATF6) pathways^37–39^. The levels of the downstream molecules in these pathways (PERK pathway: ATF4 and *CCAAT-enhancer-binding protein homologous protein* [*Chop*]; IRE1 pathway: *spliced form of x-box binding protein 1* [*Xbp1s*]; and ATF6 pathway: *binding immunoglobulin protein* [*Bip*]) were not elevated in WT IDS- or IDS mutant-expressing cells (Fig. S1). Two types of precursor IDS mutants (A85T and R468Q) were destabilized and rapidly degraded by the proteasome pathway (Fig. 3). These results indicate that IDS mutants are rapidly degraded without activation of the UPR pathways.

### WT IDS receives proteolytic cleavage at the Golgi apparatus

**Fig. 2:**
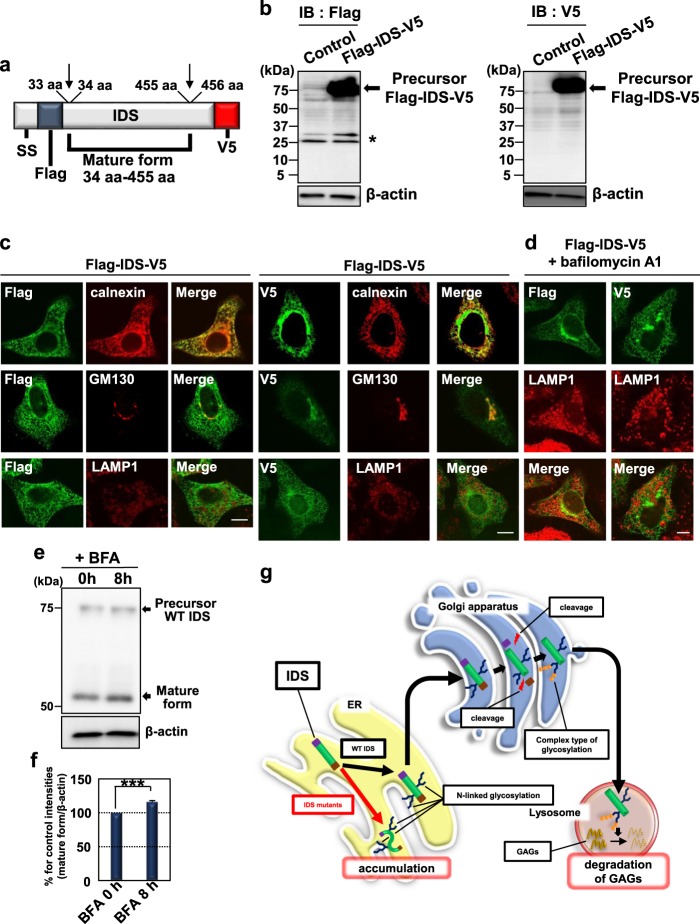
WT IDS is subjected to proteolytic cleavage in the Golgi apparatus. **a** Schema of IDS tagged with Flag (N-terminus) and V5 (C-terminus). Arrows indicate the cleavage sites. ss: signal sequence. **b** Western blotting analysis in HeLa cells expressing Flag-IDS-V5 (*N* = 3). Anti-Flag antibodies could not detect N-terminal fragments (5 kDa) generated by the cleavage (left panel). The C-terminal fragments (11 kDa) were also not detected by anti-V5 antibodies (right panel). Asterisk: nonspecific bands. **c** Immunofluorescence staining analysis using HeLa cells expressing Flag-IDS-V5. Immunoreactivities of precursor IDS were found in the calnexin-positive ER and GM130-positive *cis-*Golgi apparatus but not in the LAMP1-positive lysosome. Bars: 10 µm. **d** Immunofluorescence staining analysis using HeLa cells expressing Flag-IDS-V5. Cells were treated with 100 nM bafilomycin A1 (lysosomal protease inhibitor) for 12 h. Precursor IDS detected by anti-Flag and -V5 antibodies were not observed in lysosomes. Bar: 10 µm. **e** Western blotting analysis in HeLa cells expressing WT IDS (*N* = 3). The processed mature forms increased by treatment of cells with 1 ng/mL brefeldin A (BFA) for 8 h. **f** Quantification of relative protein levels of mature forms of IDS in (**e**) (mean ± SD, *N* = 3, Student’s *t*-test, ***P < 0.001). **g** Putative model of glycosylation and proteolytic cleavage of IDS. Precursor IDS is modified by glycosylation in the ER and Golgi apparatus. The glycosylated IDS is subjected to proteolytic cleavage in the Golgi apparatus and subsequently moves into the lysosome as mature forms to degrade GAGs. In contrast, IDS mutants are modified by glycosylation in the ER but accumulate in the ER. GAGs glycosaminoglycans

We constructed expression vector of IDS tagged with Flag and V5 at the N- and C-terminus, respectively (Fig. 2a). Western blotting using HeLa cells expressing Flag-IDS-V5 detected precursor WT IDS by anti-Flag or -V5 antibodies (Fig. 2b). The N-terminal (approximately 5 kDa detected by anti-Flag antibody) and C-terminal fragments (approximately 11 kDa detected by anti-V5 antibody) were not observed in those cells. This result suggests that the generated N- and C-terminal fragments may be rapidly degraded after cleavage. Thus, anti-Flag and -V5 antibodies exclusively detect the precursor WT IDS. Immunoreactivities of precursor WT IDS detected by anti-Flag and -V5 antibodies were found in the calnexin-positive ER and GM130-positive *cis-*Golgi apparatus (Fig. 2c). These immunoreactivities did not overlap with those of LAMP1 (lysosomal marker). We treated cells with lysosomal protease inhibitor bafilomycin A1 to prevent lysosomal degradation of any precursor WT IDS (Fig. 2d). The precursor WT IDS was not observed in the lysosome by treatment with bafilomycin A1, although the degradation of p62 (one of the substrates of lysosomal proteases^40^) was blocked by treatment with bafilomycin A1 (Fig. S2).

We then assessed whether WT IDS is subjected to proteolytic cleavage by proteases derived from the Golgi apparatus by using brefeldin A (BFA). BFA promotes Golgi disassembly and the retrograde transportation of Golgi components to the ER. The mature WT IDS levels increased after treatment of cells with BFA (Figs. 2e, f), suggesting that WT IDS is cleaved by Golgi apparatus-derived proteases. Together with the results presented in Fig. 1, we concluded that precursor IDS is subjected to proteolytic cleavage in the Golgi apparatus and subsequently moves into the lysosome as mature forms (Fig. 2g).

### IDS mutants are degraded by the ERAD pathway

**Fig. 3:**
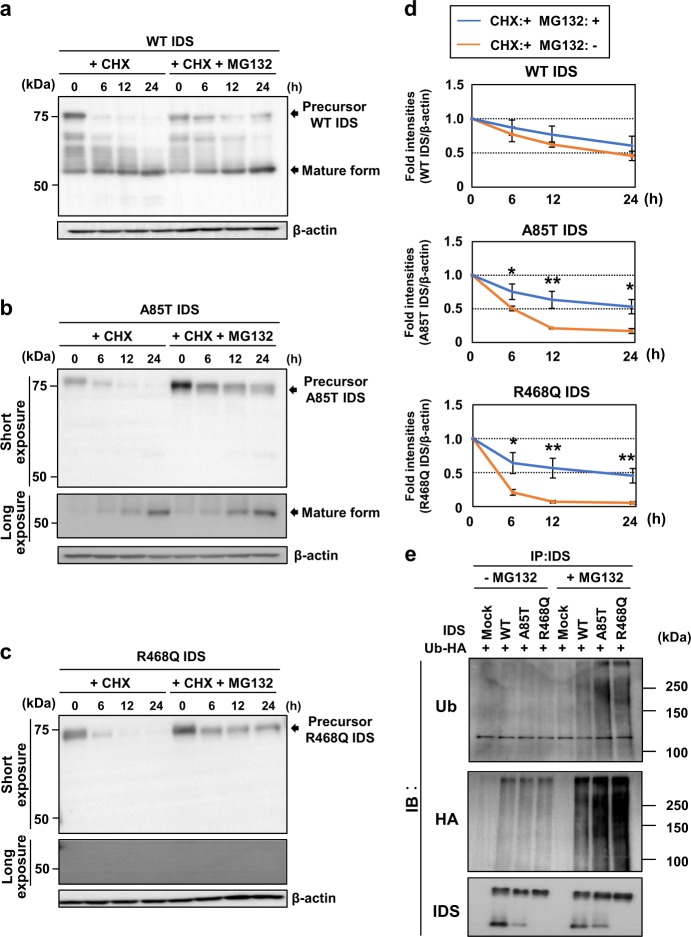
IDS mutants are unstable and are degraded by the ERAD pathway. **a**–**c** Western blotting analysis of IDS in HeLa cells expressing **a** WT IDS (*N* = 3), **b** A85T IDS (*N* = 3) and **c** R468Q IDS (*N* = 3). Cells were treated with 50 mg/mL cycloheximide (CHX) and 50 µM MG132 for the indicated times. The treatment with MG132 dramatically increased the amounts of the precursor mutants. **d** Quantification of relative protein levels of precursor WT IDS (upper), A85T IDS (middle) and R468Q IDS (lower) in **a**–**c** (mean ± SD, *N* = 3, Student’s *t*-test, *P < 0.05, **P < 0.01). **e** Immunoprecipitation assay using HeLa cells expressing WT IDS or IDS mutants and ubiquitin tagged with HA (*N* = 3). The ubiquitinated IDS mutants were detected as smeared bands and the intensities of these bands increased when cells were treated with 10 µM MG132. The intensities of the smearing bands for IDS mutants were higher than that of WT IDS. Anti-HA antibodies also detected higher intensities of ubiquitinated smeared bands of IDS mutants than that of WT IDS. Mock indicates empty vector. Ub: ubiquitin

To assess the stability of IDS, we treated HeLa cells expressing WT IDS with cycloheximide (CHX) to inhibit protein synthesis and then monitored the degradation process (Figs. 3a, d). Precursor WT IDS was observed to gradually decrease and was hardly detected by treatment with CHX for 24 h. In contrast, mature forms increased after the treatment. The amounts of precursor WT IDS slightly increased at 6, 12 and 24 h by treatment with the proteasome inhibitor MG132. The amounts of the mature forms were not significantly affected by MG132 treatment. These results suggest that WT IDS is comparatively stable and the majority of the precursor form is subjected to proteolytic cleavage to generate the mature forms with only a small amount degraded by the proteasome. Two types of precursor IDS mutants (A85T and R468Q) disappeared when cells were treated with CHX for 24 h (Figs. 3b-d). Treatment with MG132 drastically increased the amounts of these precursor mutants. The amounts of the mature forms of A85T IDS were not significantly affected by MG132 treatment (see the long exposure column for mature forms of A85T IDS). The mature forms of R468Q IDS did not appear when cells were treated with MG132. These results indicate that precursor A85T IDS and R468Q IDS are unstable and rapidly degraded by the proteasome pathway.

We further investigated the ubiquitination of precursor IDS by performing immunoprecipitation assay using HeLa cells co-transfected with WT IDS or IDS mutants and ubiquitin tagged with HA (Fig. 3e). Smeared bands of ubiquitinated IDS were barely observed in cells transfected with WT IDS or IDS mutants. Ubiquitinated smeared bands increased by treatment with MG132. Notably, the amounts of ubiquitinated IDS mutants dramatically increased, compared with that of WT IDS. Anti-HA antibodies also detected stronger intensities of ubiquitinated smeared bands for the IDS mutants, compared with that of WT IDS. In summary, precursor IDS mutants are susceptible to degradation by ERAD.

### IDS mutants are degraded via ERAD-related ubiquitin E3 ligase HRD1

**Fig. 4:**
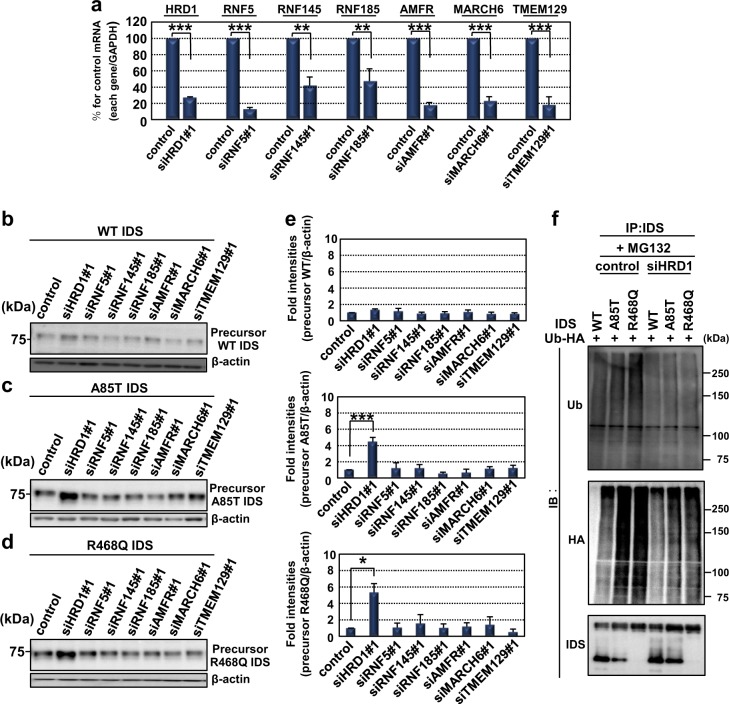
IDS mutants are degraded by the ERAD system through ubiquitin E3 ligase HRD1. **a** Quantitative PCR analysis of ERAD-related ubiquitin E3 ligases in HeLa cells expressing WT IDS. Cells were transfected with a set of siRNA#1 targeting each ubiquitin E3 ligase. The expression of each E3 ligase was downregulated by the knockdown using siRNA#1 (mean ± SD, *N* = 3, Student’s *t*-test, **P < 0.01, ***P < 0.001). **b**–**d** Western blotting analysis of IDS in HeLa cells expressing (**b**) WT IDS (*N* = 3), **c** A85T IDS (*N* = 3) and (**d**) R468Q IDS (*N* = 3). **e** Quantification of relative protein levels of precursor WT IDS (upper), A85T IDS (middle) and R468Q IDS (lower) in **b**–**d** (mean ± SD, *N* = 3, ANOVA *post hoc* Bonferroni, *P < 0.05, ***P < 0.001). **f** Immunoprecipitation assay using HeLa cells expressing WT IDS or IDS mutants and ubiquitin tagged with HA (*N* = 3). A decrease in the intensities of ubiquitinated smeared bands for IDS mutants was observed in HRD1-knockdown cells treated with 10 µM MG132. The intensities of the smeared bands detected by the anti-HA antibody were also decreased in MG132-treated HRD1-knockdown cells

To identify the ubiquitin E3 ligase responsible for the degradation of IDS mutants by ERAD, we selected major seven ER-resident ubiquitin E3 ligases established the involvement in ERAD^41,42^. The expression levels of ERAD-related ubiquitin E3 ligases we selected were suppressed from 0.15-fold to 0.45-fold in each cell transfected with two distinct sets of siRNAs (#1 and #2) and WT IDS (Figs. 4a, S3a). The level of precursor WT IDS did not show significant differences among cells transfected with two distinct siRNAs (#1 and #2) targeting each ubiquitin E3 ligase (Figs. 4a, b, e, S3a, S4a, d). In contrast, the amounts of both precursor A85T and R468Q IDS were found to only increase in cells transfected with siRNA (#1 and #2) targeting HRD1 (Figs. 4c-e, S3b, c, S4b-d). In the following experiments, we selected siHRD1#1 because it more effectively suppressed the expression of HRD1 than that of siHRD1#2. The ubiquitinated smeared bands of IDS mutants in MG132-treated cells transfected with ubiquitin tagged with HA decreased in cells transfected with siRNA targeting HRD1. The smeared bands of IDS mutants detected by anti-HA antibodies also decreased in HRD1-knockdown cells. These data indicate that both A85T and R468Q IDS mutants that accumulate in the ER are degraded by ERAD system mainly through ubiquitination by HRD1.

### HRD1-knockdown causes translocation of mutant A85T IDS from the ER to the lysosome and partial recovery of its enzyme activities

**Fig. 5:**
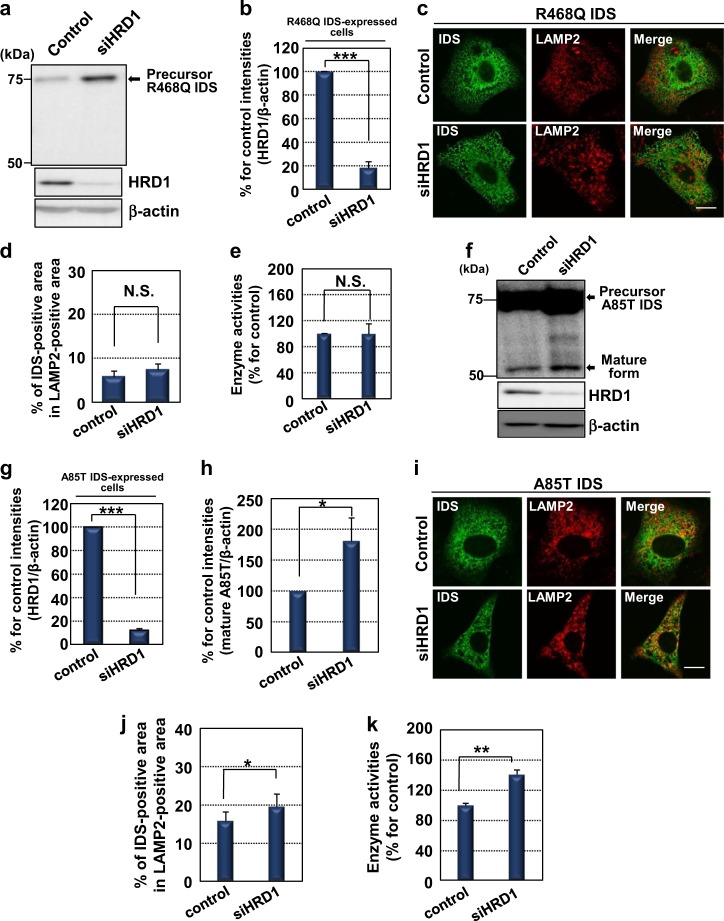
Attenuated mutant A85T IDS translocates to the lysosome and recovers its enzyme activities by HRD1-knockdown. **a** Western blotting analysis of IDS in HeLa cells expressing R468Q IDS (*N* = 3). Cells were transfected with non-targeting siRNA or siRNA targeting HRD1. **b** Quantification of relative protein levels of HRD1 in **a** (mean ± SD, *N* = 3, Student’s *t*-test, ***P < 0.001). **c** Immunofluorescence staining analysis of IDS in HeLa cells expressing R468Q IDS. Bar: 10 µm. **d** Merge ratio for the immunoreactivities of R468Q IDS and LAMP2 in **c** (mean ± SD, *N* = 7). **e** Enzyme activities of R468Q IDS in HeLa cells expressing R468Q IDS. Cells were transfected with non-targeting siRNA or siRNA targeting HRD1 (mean ± SD, *N* = 3). **f** Western blotting analysis of IDS in HeLa cells expressing A85T IDS (*N* = 3). Cells were transfected with non-targeting siRNA or siRNA targeting HRD1. **g** Quantification of relative protein levels of HRD1 in **f** (mean ± SD, *N* = 3, Student’s *t*-test, ***P < 0.001). **h** Quantification of relative protein levels of mature forms of A85T IDS in **f** (mean ± SD, *N* = 3, Student’s *t*-test, *P < 0.05). **i** Immunofluorescence staining analysis of IDS in HeLa cells expressing A85T IDS. Bar: 10 µm. **j** Merge ratio for the immunoreactivities of A85T IDS and LAMP2 in **i** (mean ± SD, *N* = 7, Student’s *t*-test, *P < 0.05). **k** Enzyme activities of A85T IDS in HeLa cells expressing A85T IDS. Cells were transfected with non-targeting siRNA or siRNA targeting HRD1 (mean ± SD, *N* = 3, Student’s *t*-test, **P < 0.01)

Previous studies have suggested that several mutant proteins (CFTR and GCase) in the ER are transported to their correct locations by inhibiting degradation^29,43,44^. Thus, we checked the processing of R468Q IDS in HRD1-knockdown cells by western blotting analysis. The protein level of HRD1 was substantially downregulated by siRNA targeting HRD1 (Figs. 5a, b). The processed mature forms of R468Q IDS were not observed in the knockdown cells, although the amount of the precursor forms increased (Fig. 5a). The immunoreactivities of R468Q IDS did not overlap with that of LAMP2 in both cells transfected with non-targeting siRNA or siRNA targeting HRD1 (Figs. 5c, d). Enzyme activities of R468Q IDS did not significantly recover by knockdown of HRD1 (Fig. 5e). The amount of the processed mature forms of A85T IDS slightly increased in HRD1-knockdown cells (Figs. 5f-h). The immunoreactivities of A85T IDS partially overlapped with those of LAMP2 (Fig. 5i). Colocalization was observed to increase in HRD1-knockdown cells (Fig. 5j). Enzyme activities were also significantly recovered by knockdown of HRD1 (Fig. 5k). In conclusion, inhibited degradation of A85T IDS by HRD1-knockdown improves translocation to the lysosome and its enzyme activities, but not those of R468Q IDS.

### ERdj3-knockdown recovers the impaired translocation and enzyme activities of A85T IDS

**Fig. 6:**
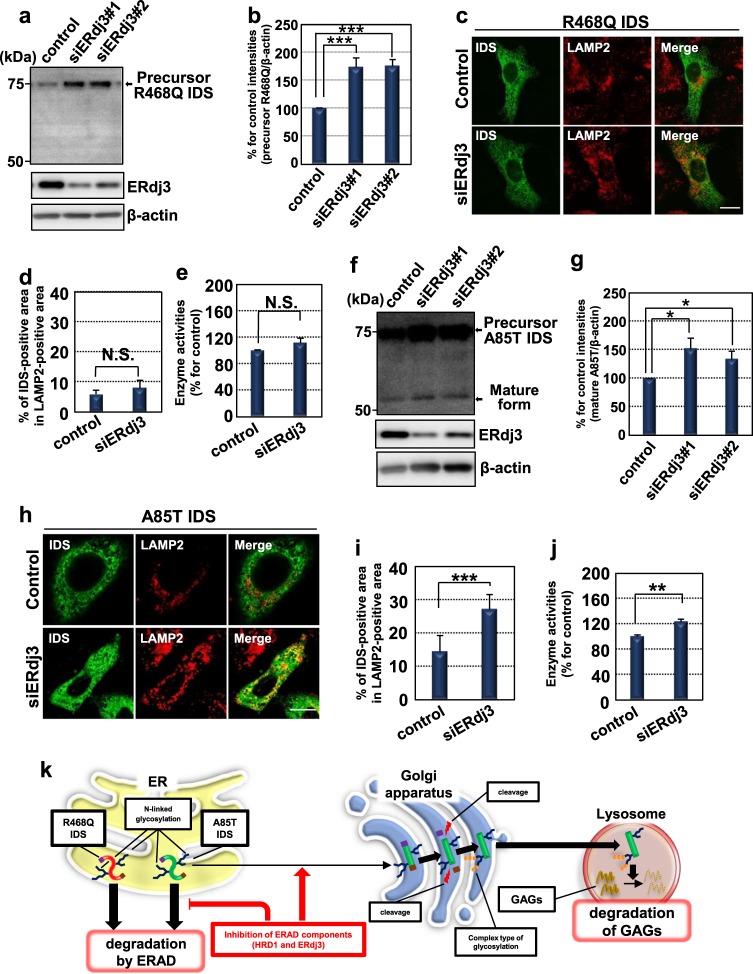
ERdj3-knockdown recovers the impaired translocation and enzyme activities of A85T IDS. **a** Western blotting analysis of IDS in HeLa cells expressing R468Q IDS (*N* = 3). Cells were transfected with non-targeting siRNA or siRNAs (#1 or #2) targeting ERdj3. **b** Quantification of relative protein levels of precursor R468Q IDS in **a** (mean ± SD, *N* = 3, Student’s *t*-test, ***P < 0.001). **c** Immunofluorescence staining analysis of IDS in HeLa cells expressing R468Q IDS. Bar: 10 µm. **d** Merge ratio for the immunoreactivities of R468Q IDS and LAMP2 in **c** (mean ± SD, *N* = 7). **e** Enzyme activities of R468Q IDS in HeLa cells expressing R468Q IDS. Cells were transfected with non-targeting siRNA or siRNA targeting ERdj3 (mean ± SD, *N* = 3). **f** Western blotting analysis of IDS in HeLa cells expressing A85T IDS (*N* = 3). Cells were transfected with non-targeting siRNA or siRNAs (#1 or #2) targeting ERdj3. **g** Quantification of relative protein levels of mature forms of A85T IDS in **f** (mean ± SD, *N* = 3, Student’s *t*-test, *P < 0.05). **h** Immunofluorescence staining analysis of IDS in HeLa cells expressing A85T IDS. Bar: 10 µm. **i** Merge ratio for the immunoreactivities of A85T IDS and LAMP2 in **h** (mean ± SD, *N* = 7, Student’s *t*-test, ***P < 0.001). **j** Enzyme activities of A85T IDS in HeLa cells expressing A85T IDS. Cells were transfected with non-targeting siRNA or siRNA targeting ERdj3 (mean ± SD, *N* = 3, Student’s *t*-test, **P < 0.01). **k** Putative model of the strategy for recovering the enzyme activities of IDS mutants. Attenuated mutant A85T IDS and severe mutant R468Q IDS accumulate in the ER followed by degradation by ERAD. A85T IDS is partially transported to the Golgi apparatus by inhibiting its degradation mediated by the HRD1- or ERdj3-related ERAD system. A85T IDS transported to the Golgi apparatus undergoes proteolytic cleavage followed by transport to the lysosome to degrade GAGs

An ERAD component ERdj3 is responsible for the delivery of GCase (lysosomal enzyme) mutant to the ERAD system^29,45^. ERdj3 was attenuated at the mRNA and protein levels in knockdown cells expressing R468Q IDS using two distinct siRNAs (#1 and #2) (Figs. 6a, S5a, b). ERdj3-knockdown did not affect the proteolytic cleavage of R468Q IDS even though increases of the precursor forms were observed (Figs. 6a, b). In the following experiments, we selected siERdj3#1 because it more effectively suppressed the expression of ERdj3 than that of siERdj3#2. The translocation of R468Q IDS to the lysosome and its enzyme activities did not improve in ERdj3-knockdown cells (Figs. 6c–e). The knockdown of ERdj3 using two distinct siRNAs (#1 and #2) slightly promoted the cleavage of A85T IDS (Figs. 6f, g, S5c, d). The translocation of A85T IDS to the lysosome and its enzyme activities were also recovered by the knockdown of ERdj3 (Figs. 6h–j). The effect of the double-knockdown (HRD1 and ERdj3) on the generation of mature forms of IDS mutants was not augmented, compared with that of the HRD1-knockdown (Fig. S6). Taken together with the HRD1-knockdown experiments, the impaired localization and enzyme activities of the attenuated mutant A85T IDS are partially rescued by inhibition of ERAD components, although this strategy is ineffective for the severe mutant R468Q IDS (Fig. 6k).

A previous study reported that pharmacological chaperone Δ-unsaturated 2-sulfouronic acid-*N*-sulfoglucosamine (D2S0) improves the function of IDS in fibroblasts derived from MPS II patients^46^. The treatment of HRD1- or double-knockdown HeLa cells with D2S0 did not promote processing of R468Q (Fig. S6a) and A85T (Fig. S6b, c) IDS mutants, compared with the knockdown cells without D2S0. These results indicate that the activation of protein folding by pharmacological chaperones may not augment the ameliorating effect on the functions of IDS mutants caused by the shutdown of ERAD.

## Discussion

In the present study, we have demonstrated that IDS mutants are prone to remain in the ER. These mutants are highly unstable and are rapidly degraded by ERAD. The shutdown of ERAD by the knockdown of HRD1 or ERdj3 partially improves impaired processing, subcellular localization and enzyme activities of A85T IDS. The double-knockdown of HRD1 and ERdj3 did not synergistically promote the processing of A85T IDS. The knockdown of HRD1 downregulates the ERAD pathway. ERdj3 is an ERAD component and its knockdown also inhibits ERAD. The silencing of both these genes did not show synergistic effects on improving the processing of IDS mutants because they regulate the same pathway.

The accumulation of a GCase mutant by inhibiting its degradation has been shown to facilitate binding to ER lectin chaperone calnexin^29^, followed by accelerating the refolding. We found that IDS undergoes N-linked glycosylation, which is necessary for recognition by calnexin^47^. The refolding of GCase mutant by calnexin may be also applicable to the refolding of IDS mutants. Thus, stimulation of folding activity may augment the recovery effect on the enzyme activities of IDS mutants in the cells attenuating ERAD. However, treatment with D2S0 did not induce the activities of IDS mutants in HRD1- or double-knockdown cells (Fig. S6). Mutant proteins lose their biological functions because of rapid degradation, conformational changes and impaired cellular localization. The comprehensive adjustment of various cellular responses including protein degradation, correct folding and activation of sorting machineries may achieve the complete recovery for the functionality of mutant proteins. The point-by-point manipulations targeting these processes partially rescue the impaired functions, but are insufficient to acquire the consummate activities of mutant proteins, including IDS mutants. Simultaneous and cooperative fine tuning of the whole cellular system may be necessary for completely ameliorating the enzyme activities of IDS mutants.

In contrast to A85T IDS, the enzyme activities of R468Q IDS could not be recovered using our strategy. R468Q IDS may be more seriously misfolded than that of A85T IDS. The R468Q mutant disturbs a buried hydrogen bonding network and hydrophobic stacking interactions that stabilize several nearby loops, helical turns and β-strands. The impaired hydrophobicity may disrupt local secondary structure and lead to buried steric clashes that result in severe misfolding^16^. A large conformational change may interrupt appropriate posttranslational modifications, the binding to calnexin and recognition by sorting systems even when degradation by ERAD is inhibited. The augmentation of multiple cellular machineries for functional activation of IDS could recover the enzyme activities of R468Q IDS.

Mutations in polypeptides can disrupt disulfide bond formation, lead to aberrant glycosylation, alter the surface charge and change the hydrophobicity of proteins^48^. Those physicochemical changes result in destabilization of the native state and aggregation as misfolded proteins. These established theories suggest that IDS mutants may accumulate in the ER as misfolded proteins. Indeed, a previous study has reported the impaired conformation of IDS mutants by X-ray crystal structure analysis^16^. Disulfide bonds in IDS (C171−C184 and C422−C432 disulfide bonds) are functionally important, and mutation of these cysteines causes MPS II^49–52^. IDS mutants characterized by deficient glycosylation (N115I) and production of new glycosylation sites (T130N) also progress the pathogenesis of MPS II^16^. The L410P mutant disturbs a buried hydrogen bond and induces steric clashes. Other types of mutants generate inversion of local charges (E521K) and alteration of the surface charge (E125V). These mutations can trigger alternative conformations, leading to non-native IDS folding that will be recognized as misfolded proteins by ERAD and be degraded.

The enzyme activities of attenuated type of IDS mutant are only 0.6% higher than those IDS mutants that are classed as the severe type (Fig. 1b)^19^. However, the symptoms and life expectancies of MPS II patients suffering from the late-onset form are noticeably better, compared with those patients suffering the early-onset form^4^. We found that the shutdown of ERAD partially recovers the enzyme activities of A85T IDS by increasing approximately 1.3-fold. Although only partial recovery of enzyme activities of this mutant was achieved, various studies on MPS II patients in early- and late-onset forms indicate that only a small increase of enzyme activities drastically relieves symptoms of MPS II^4,19^. Additionally, previous reports suggest that a substantial therapeutic effect can be obtained when the enzyme activities of the mutant IDS show only a few percent improvement and are only marginally active, compared with that of WT IDS^53,54^. Therefore, our strategy for recovering the enzyme activities of the IDS mutants by inhibiting degradation of the protein has the potential to relieve symptoms suffered by MPS II patients.

In conclusion, we have demonstrated that IDS mutants that accumulate in the ER are degraded by the ERAD system. The impaired localization and enzyme activities of attenuated IDS mutants are rescued by inhibiting their degradation via knockdown of HRD1 or ERdj3. Our present study suggests a new target for ameliorating MPS II and shows the prospective possibilities to provide novel therapeutic strategies that may prologue to achieve complete recovery of MPS II.

## Materials and methods

### Cell culture, reagents, plasmids and siRNA

HeLa cells (RRID: CVCL_0030) were maintained in Dulbecco’s modified Eagle’s medium (Gibco, Rockville, MD, USA) supplemented with 10% (v/v) heat-inactivated fetal bovine serum at 37 °C in a 5% CO_2_, 95% humidified air atmosphere. For cell treatments, 10 μM or 50 μM MG132 (Wako, Osaka, Japan), 50 mg/mL CHX (Wako), 100 nM bafilomycin A1 (Sigma, St. Louis, MO, USA), 1 ng/mL BFA (Sigma), 1 µM thapsigargin (Wako) and 0.1 µM or 10 µM Δ-unsaturated 2-sulfouronic acid-*N*-sulfoglucosamine (Iduron, Alderley Edge, UK) were used.

The pcDNA3.1(+) vectors expressing WT human IDS and human IDS mutants (R48P, A85T, W337R, Q531X, P86L, S333L and S349I) were constructed previously^19^. The vector with the complementary DNA (cDNA) of IDS mutant (R468Q) inserted was a kind gift from Dr. Voraratt Champattanachai (Chulabhorn Research Institute, Bangkok, Thailand). The cDNA was recloned into the pcDNA3.1(+) vector. The pcDNA3.1(+) vector expressing FLAG-IDS-V5 was generated by PCR. Primer sequences are summarized in Supplementary Table S1. The transfections of expression vectors were performed using ScreenFect A (Wako), according to the manufacturer’s protocol. Cells transfected with expression vectors were collected 72 h after the transfection to use in the experiments.

For the knockdown of ubiquitin E3 ligases and ERdj3, HeLa cells were transfected with non-targeting siRNA (Thermo Fisher Scientific, Waltham, MA, USA), siRNA targeting for HRD1 (Dharmcon. Lafayette, CO, USA, J-007090-05-0020, Thermo Fisher Scientific, s39019), RNF5 (Thermo Fisher Scientific, s12076, s12077), RNF145 (Thermo Fisher Scientific, s45830, s45831), RNF185 (Thermo Fisher Scientific, s230675, s40665), AMFR (Thermo Fisher Scientific, s1323, s1324), MARCH6 (Thermo Fisher Scientific, s20137, s20138), TMEM129 (Thermo Fisher Scientific, s40917, s40915) or ERdj3 (Thermo Fisher Scientific, s28581, s28580) by ScreenFect A (Wako). The transfected cells were incubated for 72 h and then harvested for western blotting and immunofluorescence staining analyses.

### RNA isolation, RT-PCR and quantitative PCR

Total RNA was isolated from HeLa cells using ISOGEN (Wako) according to the manufacturer’s protocol. First-strand cDNA was synthesized from 1 μg RNA in a 20 μL reaction volume, using a random primer (Takara, Kusatsu, Japan) and Moloney murine leukemia virus reverse transcriptase (Invitrogen, Carlsbad, CA, USA). The expression levels of *Bip*, *Chop*, *Xbp1* and β-*actin* were analyzed by performing PCR in a 20 μL reaction mixture containing 0.5 μM of each primer, 0.2 mM dNTPs, three units of Taq polymerase and 10 × PCR buffer (Agilent, Santa Clara, CA, USA). The PCR conditions were as follows: 94 °C for 2 min; 25 cycles (Chop and *Xbp1*) and 20 cycles (*Bip* and β*-actin*) of 94 °C for 30 s, 55 °C for 30 s and 72 °C for 1 min. The PCR products were resolved by electrophoresis on a 4.8% acrylamide gel. Primer sequences are summarized in Supplementary Table 1. The density of each band was quantified using the CS Analyzer 4 image analysis software (ATTO, Tokyo, Japan). For quantitative PCR, the reverse-transcribed cDNA was measured by the TaqMan-based real-time PCR assay using the 2^–∆∆Ct^ method and a Light Cycler 480 system II (Roche, Basel, Switzerland).

### Protein preparation and western blotting

Proteins were extracted from HeLa cells in a cell lysis buffer containing 50 mM Tris-HCl (pH 7.5), 150 mM NaCl, 1% Triton X-100 and the Protease inhibitor cocktail Set V (Wako) at 4 °C. The lysates were incubated on ice for 45 min. After centrifugation at 15,000 *g* for 15 min, the protein concentrations of the supernatants were determined using a bicinchoninic acid assay (BCA) kit (Thermo Fisher Scientific). Equal amounts of proteins (10 μg) were used for sodium dodecyl sulfate–polyacrylamide gel electrophoresis (SDS-PAGE). For immunoblotting, the following antibodies were used: anti-β-actin (mouse IgG, 1:5000; Santa Cruz Biotechnology, Santa Cruz, CA, USA, RRID: AB_1119529), anti-Flag M2 (mouse IgG, 1:2000; Sigma, RRID: AB_439685), anti-V5 (mouse IgG, 1:2000; Thermo Fisher Scientific, RRID: AB_2556564), anti-ATF4 (rabbit IgG, 1:3000; Santa Cruz Biotechnology, RRID: AB_2058752), anti-ERdj3 (rabbit IgG, 1:3000; Proteintech, Chicago, IL, USA, RRID: AB_2094400), anti-p62 (rabbit IgG, 1:2000; Medical & Biological Laboratories, Nagoya, Japan, RRID: AB_1279301) and anti-IDS (mouse IgG, 1:2000; R & D systems, Minneapolis, MN, USA, RRID: AB_2233551). The density of each band was quantified using the CS Analyzer 4 image analysis software (ATTO). For digestion of carbohydrates, 500 U PNGase F (*N*-glycosidase F) (New England Biolabs, Ipswich, MA, USA) and 500 U Endo H (endoglycosidase H) (New England Biolabs) were mixed with cell lysates at 37 °C for 1 h in Glycobuffer (New England Biolabs) and 1% NP40. The samples were subjected to SDS-PAGE followed by western blotting analysis.

### Immunofluorescence staining

HeLa cells were grown on coverslips and fixed in 4% paraformaldehyde for 10 min. After fixation, cells were permeabilized in 0.1% Triton X-100 for 10 min, followed by treatment with 5% bovine serum albumin for 60 min. These procedures were performed at room temperature (25 °C). The following antibodies were used: anti-IDS (goat IgG, 1:100; R & D systems, RRID: AB_2123559), anti-Flag M2 (mouse IgG, 1:200; Sigma, RRID: AB_439685), anti-V5 (mouse IgG, 1:200; Thermo Fisher Scientific, RRID: AB_2556564), anti-calnexin (mouse IgG, 1:200; EMD Millipore, Billerica, MA, USA, RRID: AB_2069152), anti-calnexin (rabbit IgG, 1:200; Cell Signaling Technology, Beverly, MA, USA, RRID: AB_2228381), anti-GM130 (mouse IgG, 1:200; BD Biosciences, Franklin Lakes, NJ, USA, RRID: AB_398141), anti-LAMP1 (rabbit IgG, 1:100; Cell Signaling Technology, RRID: AB_2687579) and anti-LAMP2 (mouse IgG, 1:2000; BD Biosciences, RRID: AB_396137). Cells were visualized under a FV1000D confocal microscope (Olympus, Tokyo, Japan). The merge ratio for describing the immunoreactivities of IDS and LAMP2 were measured by using ImageJ (National Institutes of Health, Rockville, MD, USA).

### Measurement of enzyme activities

The enzyme activities of WT and mutant IDS proteins were measured according to a modified procedure^46^. Briefly, the enzyme activities of IDS were determined with sonicated cells in water using the artificial substrate 4-methylumbelliferylalpha-iduronide-2-sulfate (MU-αIdu-2S) (Toronto Research Chemicals, Ontario, Canada). Protein concentrations were determined using the BCA kit (Thermo Fisher Scientific). A total of 10 μg of protein was incubated with 20 μL of the substrate solution (1.25 mM 4MU-αIdu-2S, 0.1 M Na-acetate buffer (pH 5.0)), containing 10 mM lead-acetate and 0.02% sodium azide at 37 °C for 4 h in a dark room. Next, 40 μL of McIlvain’s buffer [0.4 M Na-phosphate containing 0.2 M citrate, (pH 4.5)] and 250 ng/10 μL purified recombinant human IDUA (R&D systems) were added to the incubation samples, which were then incubated for an additional 24 h at 37 °C. Finally, the reaction was terminated by the addition of 200 μL of the stop buffer [0.5 M NaHCO_3_, 0.5 M Na_2_CO_3_ buffer (pH 10.7)] to the mixture. The liberated fluorescence of the 4-methylumbelliferone was then measured with a Tristar LB941 spectrofluorophotometer (Berthold Technologies, Bad Wildbad, Germany) at 355 nm excitation and 460 nm emission.

### Immunoprecipitation assay

HeLa cells transfected with vectors expressing IDS and HA tagged with ubiquitin were lysed in cell lysis buffer [50 mM Tris-HCl (pH 7.5), 150 mM NaCl, 1% Triton X-100, 5 mM EDTA and Protease inhibitor cocktail Set V (Wako)] for 20 min. Supernatants were incubated with anti-IDS (goat IgG, 1:2000; R & D Systems, RRID: AB_2123559) antibodies and Protein G Agarose Beads (Invitrogen) at 4 °C for 4 h. The beads were rinsed five times with a wash buffer [20 mM Tris-HCl (pH 7.5), 150 mM NaCl, 0.1% Triton X-100]. Immunoprecipitates were boiled with SDS-PAGE sample buffer, followed by western blotting using the antibodies: anti-IDS (mouse IgG, 1:2000; R & D Systems, RRID: AB_2233551), anti-ubiquitin (mouse IgG, 1:1000; Cell Signaling Technology, RRID: AB_10998070) and anti-HA (rabbit IgG, 1:1000; Cell Signaling Technology, RRID: AB_1549585).

### Statistical analysis

Statistical comparisons were made using an unpaired Student’s *t*-test (between two samples) and analysis of variance (ANOVA) followed by Bonferroni *post hoc* test (among multiple samples). The statistical significance of a difference was determined on the basis of a *P* < 0.05. Here, *P*-values <0.05, 0.01 or 0.001 are presented as *P < 0.05, **P < 0.01 or ***P < 0.001, respectively. Double-blind analyses were applied for all quantifications.

## Electronic supplementary material

Figure Legends Fig. S1,Fig. S2,Fig. S3, Fig. S4,Fig. S5,Fig. S6, Table S1

Fig. S1,Fig. S2,Fig. S3, Fig. S4,Fig. S5,Fig. S6, Table S1

Supplementary figure legends
