# Supplementary material for: Shutdown of ER-associated degradation pathway rescues functions of mutant iduronate 2-sulfatase linked to mucopolysaccharidosis type II
Source: Cell Death Dis. 2018 Jul 24;9(8):808. doi: 10.1038/s41419-018-0871-8 (PMC6057917; doi:10.1038/s41419-018-0871-8)
Supplement: Supplementary file 1 — Figure Legends Fig. S1,Fig. S2,Fig. S3, Fig. S4,Fig. S5,Fig. S6, Table S1 [file 41419_2018_871_MOESM1_ESM.pdf]

Supplementary Information

Supplementary Figure Legends

Supplementary Fig. 1. Unfolded protein response is not activated by the transient expression of IDS mutants.

a Western blotting ($N = 3$) and RT-PCR ($N = 3$) analysis of ATF4, *Bip*, *Chop* and *Xbp1* in HeLa cells expressing WT IDS or IDS mutants. The expression levels of ATF4, *Bip* and *Chop* were induced by treatment with 1 μM thapsigargin (Tg) (ER stressor). *Unspliced form of Xbp1* (*Xbp1u*) was spliced and *spliced form of Xbp1* (*Xbp1s*) was generated under these conditions. In contrast, induction of these proteins and genes (ATF4, *Bip*, *Chop* and *Xbp1s*) was not observed in cells expressing WT IDS or IDS mutants. **b** Quantification of the relative protein level of ATF4 in (**a**) (mean ± SD, $N = 3$, Student's *t*-test, $^{**}P < 0.01$). **c** Quantification of the relative mRNA level of *Bip* in (**a**) (mean ± SD, $N = 3$, Student's *t*-test, $^{*}P < 0.05$). **d** Quantification of the relative mRNA level of *Chop* in (**a**) (mean ± SD, $N = 3$, Student's *t*-test, $^{***}P < 0.001$). **e** Quantification of the relative mRNA level of *Xbp1s* in (**a**) (mean ± SD, $N = 3$, Student's *t*-test, $^{*}P < 0.05$).

Supplementary Fig. 2. Treatment with bafilomycin A1 effectively inhibits the activities of lysosomal proteases.

a Western blotting analysis of p62 (substrate of lysosomal proteases) in HeLa cells expressing Flag-IDS-V5 (*N* = 3). Cells were treated with 100 nM bafilomycin A1 for 12 h. **b** Quantification of the relative protein level of p62 in (**a**) (mean ± SD, *N* = 3, Student's *t*-test, ***$P < 0.001$).

Supplementary Fig. 3. Two distinct siRNAs targeting each ubiquitin E3 ligase effectively block the expression of the target E3 ligases.

a-c Quantitative PCR analysis of ERAD-related ubiquitin E3 ligases in HeLa cells expressing (**a**) WT IDS (*N* = 3), (**b**) A85T IDS (*N* = 3) and (**c**) R468Q IDS (*N* =3). Cells were transfected with non-targeting siRNA or siRNA targeting each E3 ligase. The set of siRNA#1 (WT IDS) and a set of siRNA#1 or #2 (A85T and R468Q IDS) were used for the knockdown (mean ± SD, *N* = 3, Student's *t*-test, *$P < 0.05$, **$P < 0.01$, ***$P < 0.001$).

Supplementary Fig. 4. Degradation of the IDS mutants is inhibited by knockdown of the ubiquitin E3 ligase HRD1.

a-**c** Western blotting analysis of IDS in HeLa cells expressing (**a**) WT IDS (*N* = 3), (**b**) A85T IDS (*N* = 3) and (**c**) R468Q IDS (*N* = 3). Cells were transfected with non-targeting siRNA or siRNA#2 targeting each E3 ligase. **d** Quantification of relative protein levels of precursor WT IDS (upper), A85T IDS (middle) and R468Q IDS (lower) in (**a**-**c**) (mean ± SD, $N = 3$, ANOVA *post hoc* Bonferroni, ***$P < 0.001$).

Supplementary Fig. 5. Two distinct siRNAs targeting ERdj3 effectively block the expression of ERdj3.

a Quantitative PCR analysis of ERdj3 in HeLa cells expressing R468Q IDS ($N = 3$). Cells were transfected with non-targeting siRNA or siRNA targeting ERdj3 (mean ± SD, $N = 3$, Student's *t*-test, ***$P < 0.001$). The numbers indicate the percentages for control mRNA. **b** Quantification of the relative protein level of ERdj3 in (**Fig. 6a**) (mean ± SD, $N = 3$, Student's *t*-test, ***$P < 0.001$). **c** Quantitative PCR analysis of ERdj3 in HeLa cells expressing A85T IDS ($N = 3$). Cells were transfected with non-targeting siRNA or siRNA targeting ERdj3 (mean ± SD, $N = 3$, Student's *t*-test, ***$P < 0.001$). The numbers indicate the percentages for control mRNA. **d** Quantification of the

relative protein level of ERdj3 in (**Fig. 6f**) (mean ± SD, $N = 3$, Student's t-test, ***$P < 0.001$).

Supplementary Fig. 6. Double knockdown of HRD1 and ERdj3, and shutdown of ERAD combined with a pharmacological chaperone do not augment recovery effects on the functions of IDS mutants.

a Western blotting analysis of IDS in HeLa cells expressing R468Q IDS mutants ($N = 3$). Cells were transfected with non-targeting siRNA or siRNA targeting HRD1 and ERdj3. The knockdown cells were treated with 10 μM D2S0 for 48 h[46]. **b** Western blotting analysis of IDS in HeLa cells expressing A85T IDS mutants ($N = 3$). Cells were transfected with non-targeting siRNA or siRNA targeting HRD1 and ERdj3. The knockdown cells were treated with 0.1 μM D2S0 for 48 h[46]. **c** Quantification of the relative protein levels of mature forms of A85T IDS in (**b**) (mean ± SD, $N = 3$).

Supplementary Table 1.

Each specific primer set used to construct plasmids and RT-PCR.
