# Supplementary material for: Shutdown of ER-associated degradation pathway rescues functions of mutant iduronate 2-sulfatase linked to mucopolysaccharidosis type II
Source: Cell Death Dis. 2018 Jul 24;9(8):808. doi: 10.1038/s41419-018-0871-8 (PMC6057917; doi:10.1038/s41419-018-0871-8)
Supplement: Supplementary file 2 — Fig. S1,Fig. S2,Fig. S3, Fig. S4,Fig. S5,Fig. S6, Table S1 [file 41419_2018_871_MOESM2_ESM.pdf]

Supplementary Figure 1

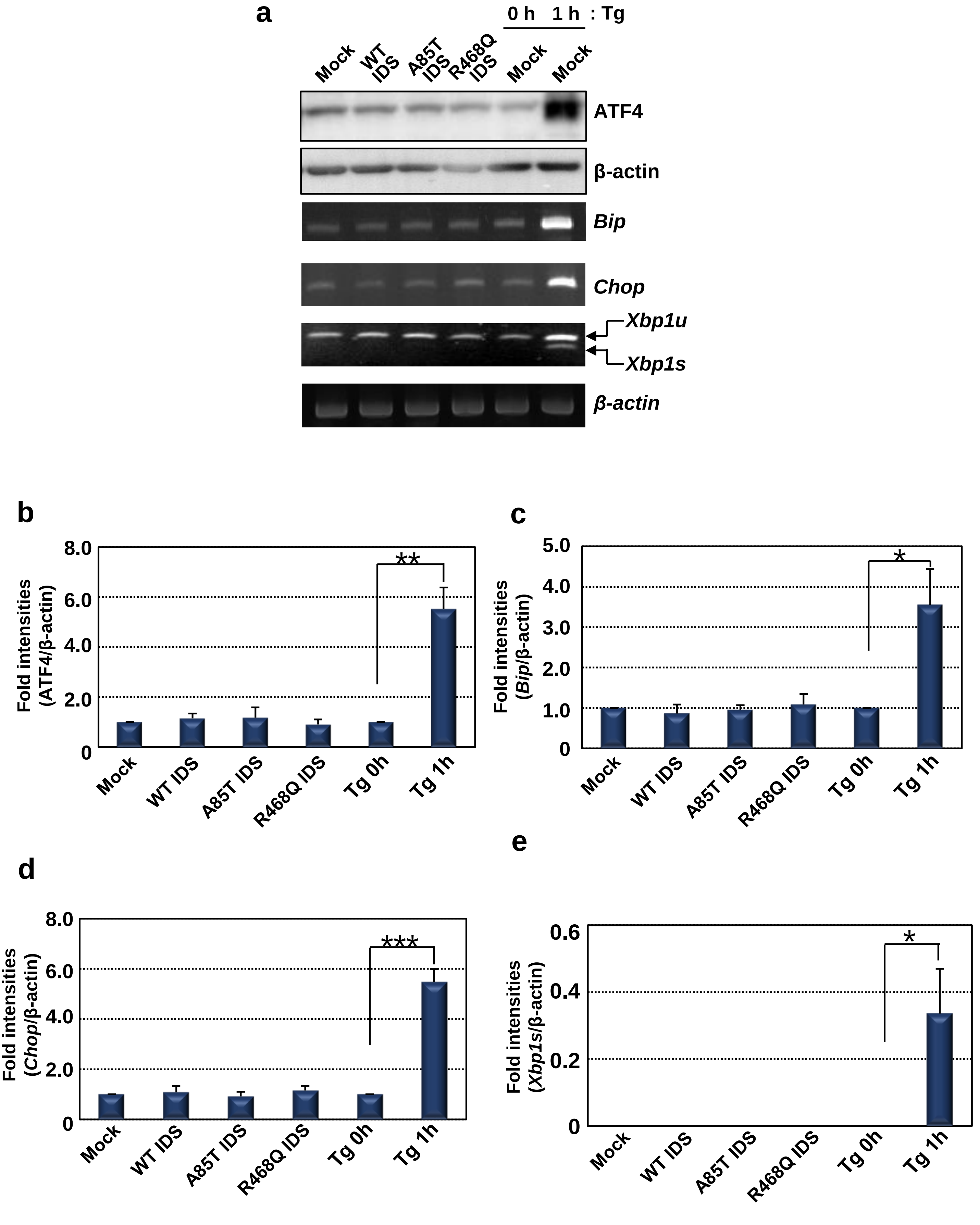

Supplementary Figure 2

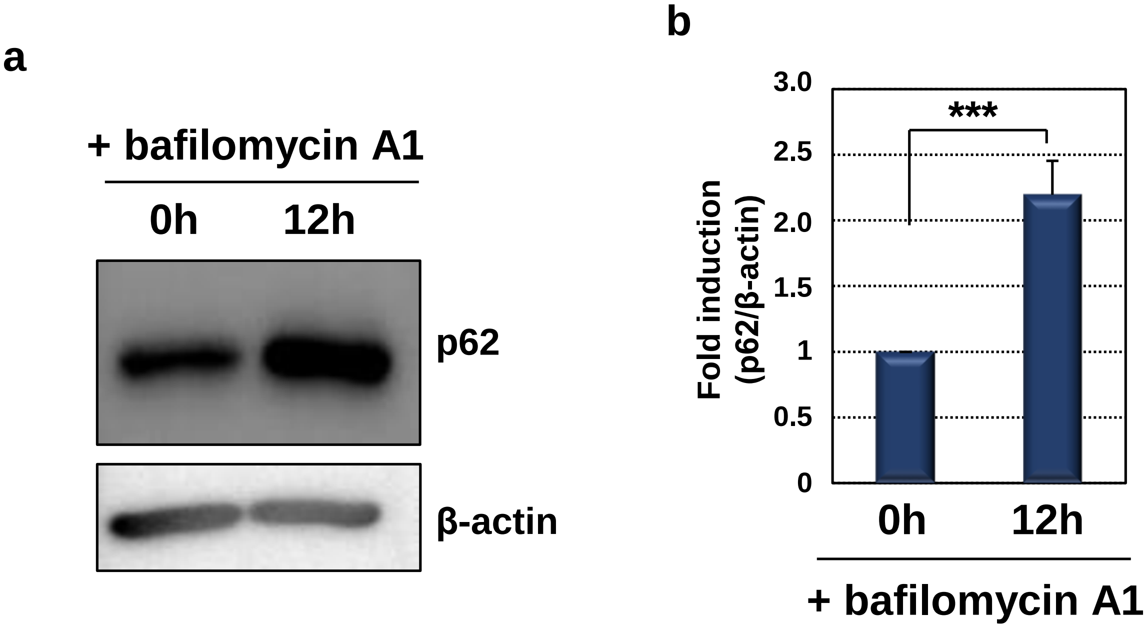

Supplementary Figure 3

a

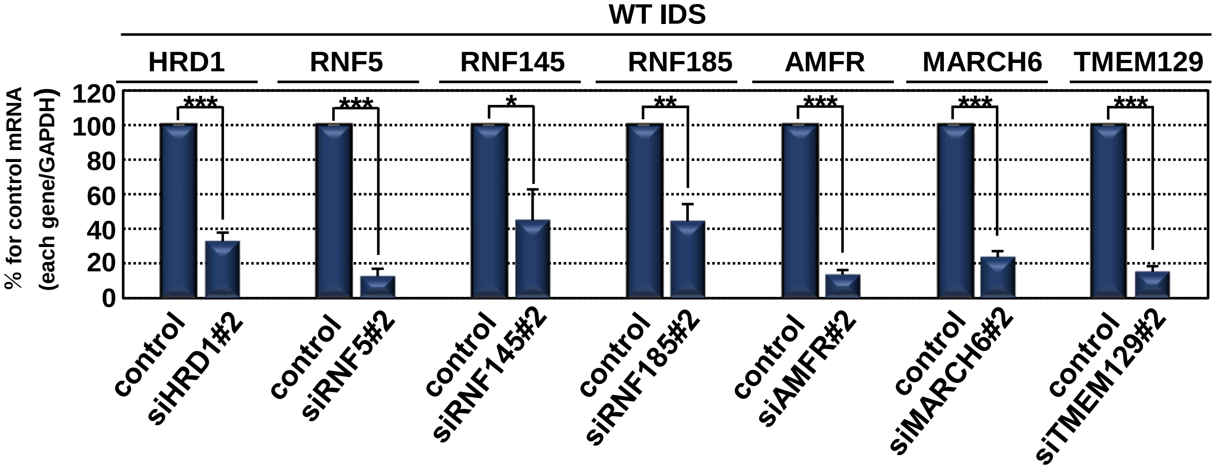

b

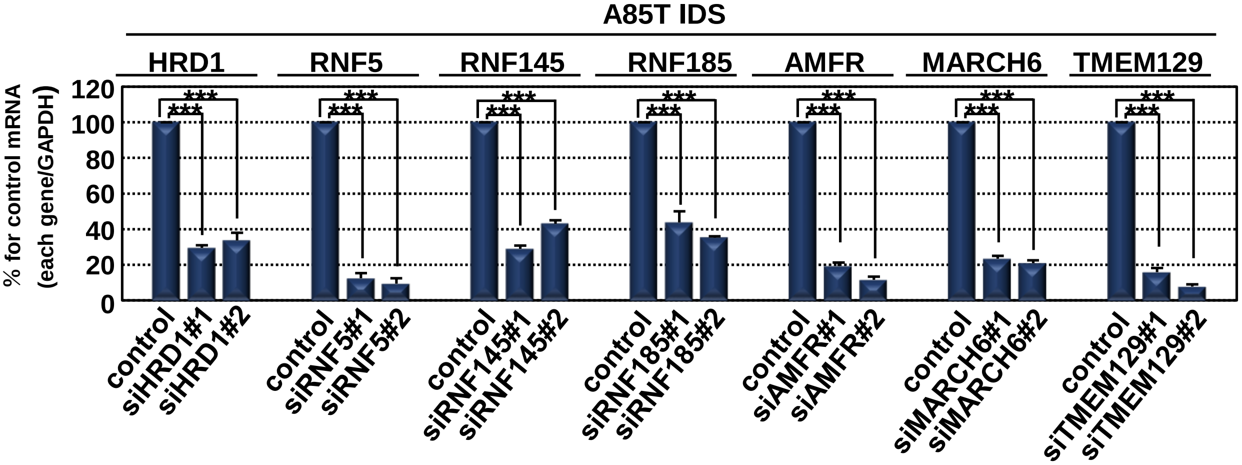

c

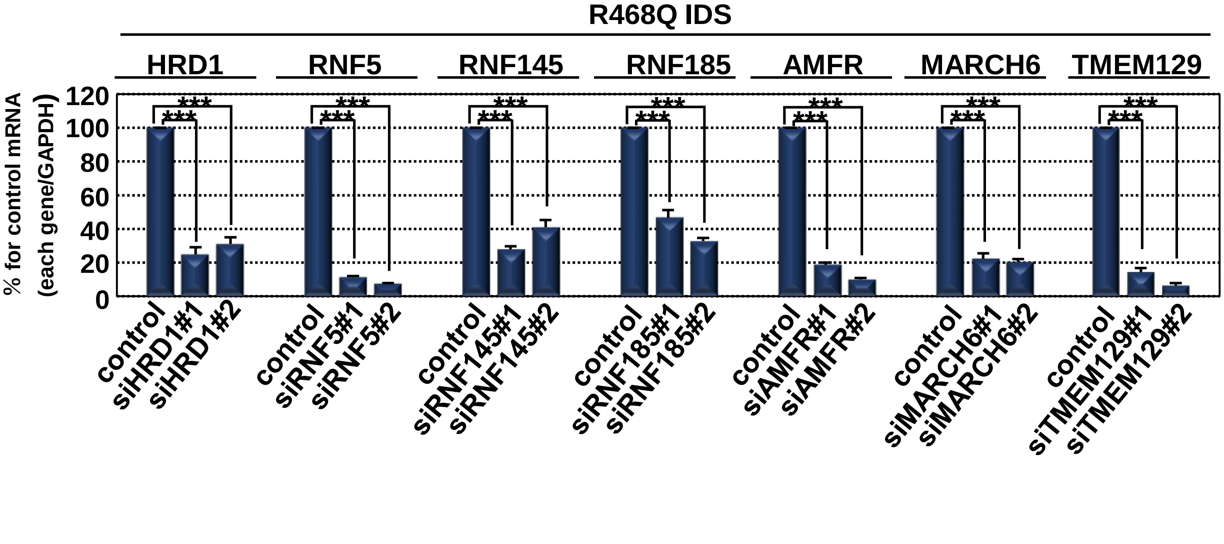

Supplementary Figure 4

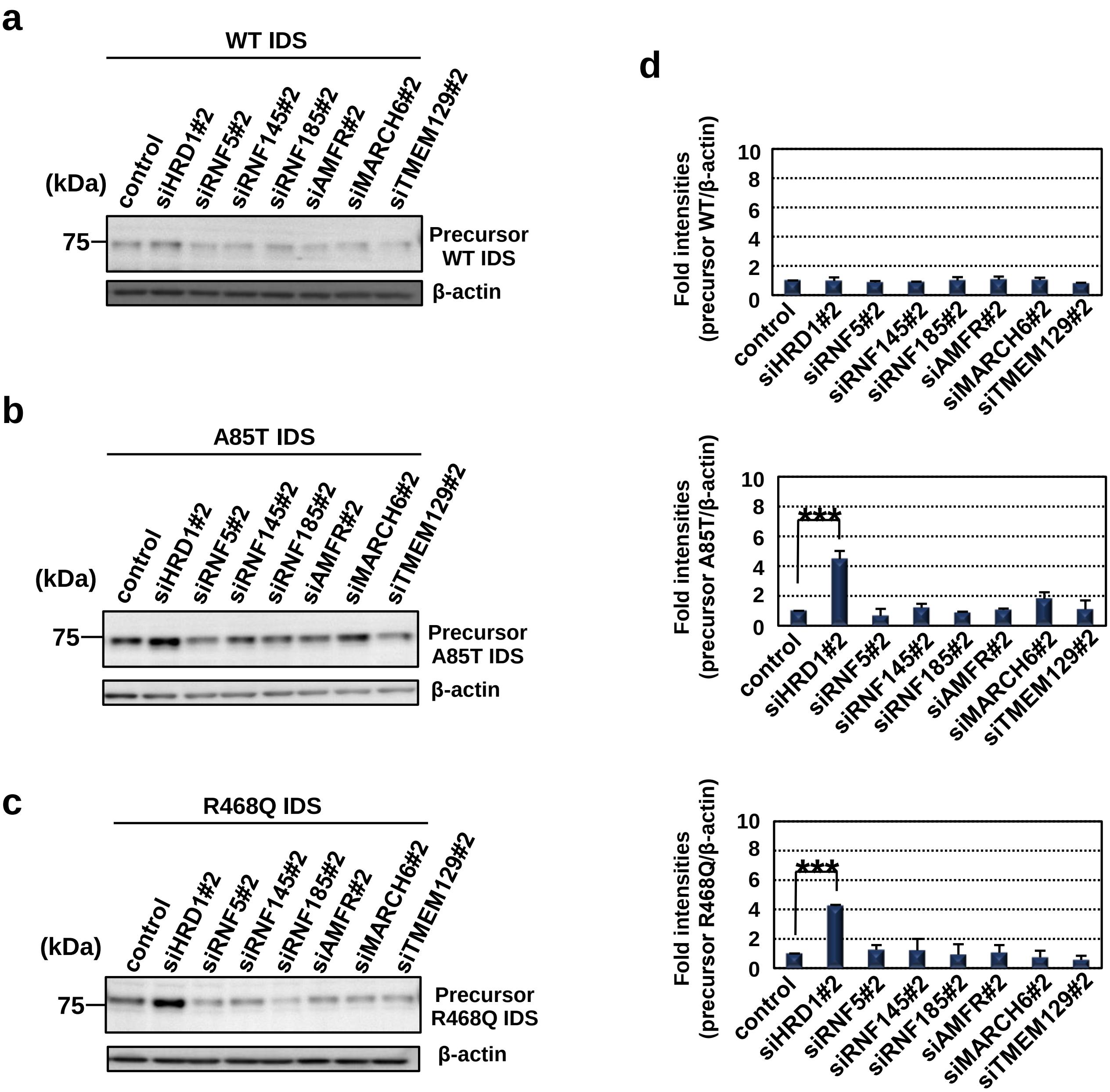

Supplementary Figure 5

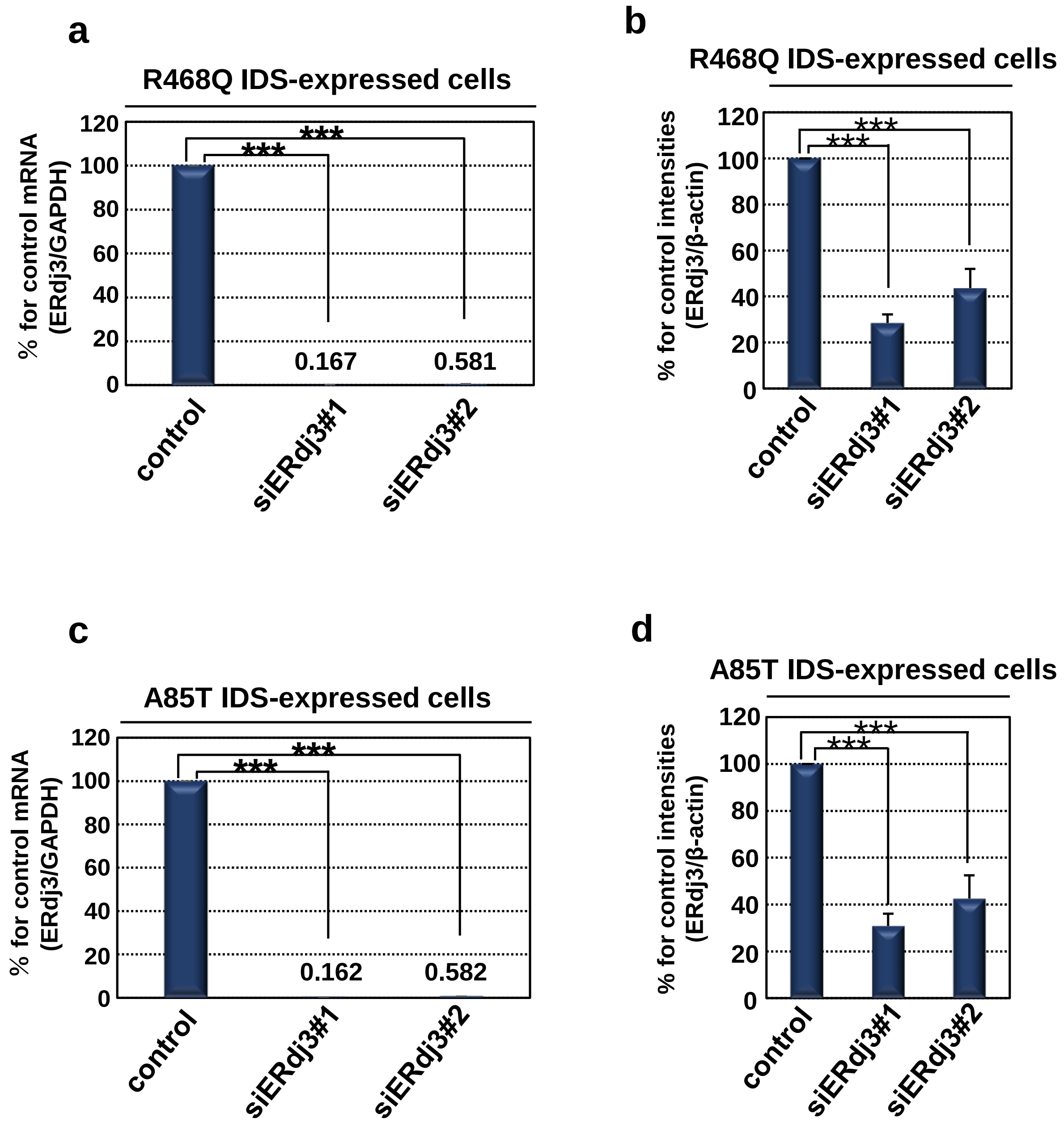

Supplementary Figure 6

a

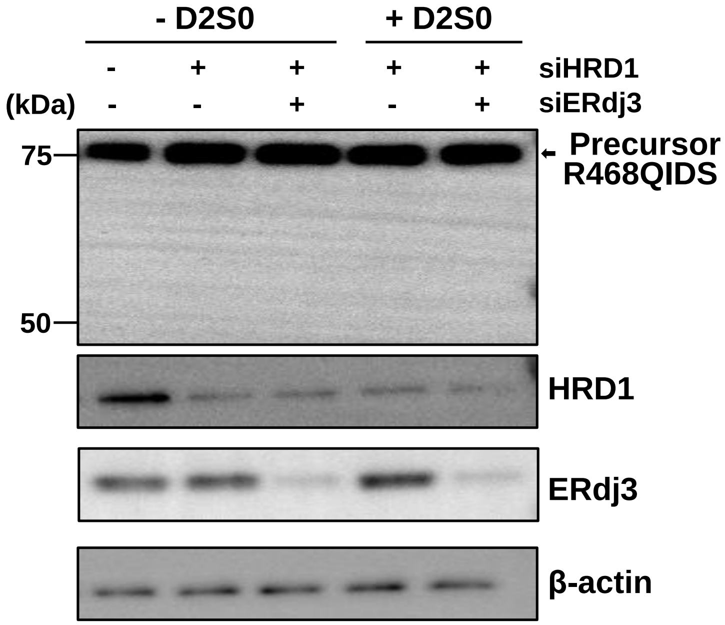

b

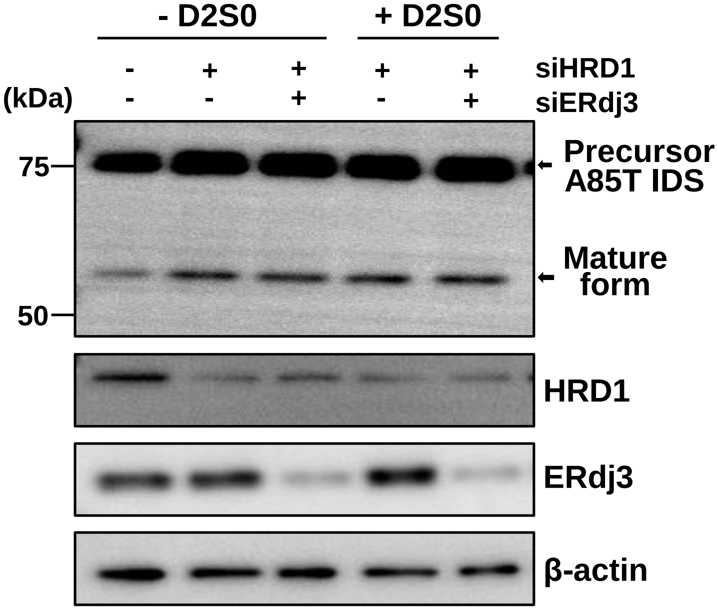

c

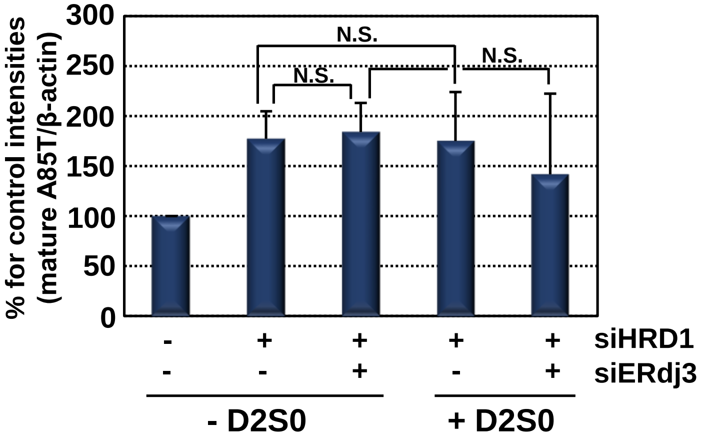

Supplementary Table 1

Gene name	Sense primer (5'-3')	Antisense primer (5'-3')
FLAG-IDS-V5 signal sequence	CTAGCCCACCATGCCGCCACCCCGGACCG	GGTACCTCCGAGGGCGACGCAGACGGAGCT
FLAG-IDS-V5	GGTACCGACTACAAGGACGACGATGACAAGTCCGAAACGCAGGCCAACTC	TCACGTAGAATCGAGACCGAGGAGAGGGTTAGGGATAGGCTTACCAGGCATCAACAACTGGA
Bip	GTTTGCTGAGGAAGACAAAAAGCTC	CACTTCCATAGAGTTTGCTGATAATTG
Chop	GTCCAGCTGGGAGCTGGAAG	CTGACTGGAATCTGGAGAG
Xbp1	CAGCGCTTGGGGATGGATGC	CCATGGGGAGATGTTCTGGA
β-actin	TCCTCCCTGGAGAAGAGCTAC	TCCTGCTTGCTGATCCACAT
