# Supplementary material for: Shutdown of ER-associated degradation pathway rescues functions of mutant iduronate 2-sulfatase linked to mucopolysaccharidosis type II
Source: Cell Death Dis. 2018 Jul 24;9(8):808. doi: 10.1038/s41419-018-0871-8 (PMC6057917; doi:10.1038/s41419-018-0871-8)
Supplement: Supplementary file 3 — Supplementary figure legends [file 41419_2018_871_MOESM3_ESM.docx]

**Supplementary Information**

**Supplementary Figure Legends**

**Supplementary Fig. 1. Unfolded protein response is not activated by the transient expression of IDS mutants.**

**a** Western blotting (*N* = 3) and RT-PCR (*N* = 3) analysis of ATF4, *Bip*, *Chop* and *Xbp1* in HeLa cells expressing WT IDS or IDS mutants. The expression levels of ATF4, *Bip* and *Chop* were induced by treatment with 1 μM thapsigargin (Tg) (ER stressor). *Unspliced form of Xbp1* (*Xbp1u*) was spliced and *spliced form of Xbp1* (*Xbp1s*) was generated under these conditions. In contrast, induction of these proteins and genes (ATF4, *Bip*, *Chop* and *Xbp1s*) was not observed in cells expressing WT IDS or IDS mutants. **b** Quantification of the relative protein level of ATF4 in (**a**) (mean ± SD, *N* = 3, Student's *t*-test, ***P* < 0.01). **c** Quantification of the relative mRNA level of *Bip* in (**a**) (mean ± SD, *N* = 3, Student's *t*-test, **P* < 0.05). **d** Quantification of the relative mRNA level of *Chop* in (**a**) (mean ± SD, *N* = 3, Student's *t*-test, ****P* < 0.001). **e** Quantification of the relative mRNA level of *Xbp1s* in (**a**) (mean ± SD, *N* = 3, Student's *t*-test, **P* < 0.05).

**Supplementary Fig. 2. Treatment with bafilomycin A1 effectively inhibits the activities of lysosomal proteases.**

**a** Western blotting analysis of p62 (substrate of lysosomal proteases) in HeLa cells expressing Flag-IDS-V5 (*N* = 3). Cells were treated with 100 nM bafilomycin A1 for 12 h. **b** Quantification of the relative protein level of p62 in (**a**) (mean ± SD, *N* = 3, Student's *t*-test, ****P* < 0.001).

**Supplementary Fig. 3. Two distinct siRNAs targeting each ubiquitin E3 ligase effectively block the expression of the target E3 ligases.**

**a**-**c** Quantitative PCR analysis of ERAD-related ubiquitin E3 ligases in HeLa cells expressing (**a**) WT IDS (*N* = 3), (**b**) A85T IDS (*N* = 3) and (**c**) R468Q IDS (*N* =3). Cells were transfected with non-targeting siRNA or siRNA targeting each E3 ligase. The set of siRNA#1 (WT IDS) and a set of siRNA#1 or #2 (A85T and R468Q IDS) were used for the knockdown (mean ± SD, *N* = 3, Student's *t*-test, **P* < 0.05, ***P* < 0.01, ****P* < 0.001).

**Supplementary Fig. 4. Degradation of the IDS mutants is inhibited by knockdown of the ubiquitin E3 ligase HRD1.**

**a**-**c** Western blotting analysis of IDS in HeLa cells expressing (**a**) WT IDS (*N* = 3), (**b**) A85T IDS (*N* = 3) and (**c**) R468Q IDS (*N* = 3). Cells were transfected with non-targeting siRNA or siRNA#2 targeting each E3 ligase. **d** Quantification of relative protein levels of precursor WT IDS (upper), A85T IDS (middle) and R468Q IDS (lower) in (**a**-**c**) (mean ± SD, *N* = 3, ANOVA *post hoc* Bonferroni, ****P* < 0.001).

**Supplementary Fig. 5. Two distinct siRNAs targeting ERdj3 effectively block the expression of ERdj3.**

**a** Quantitative PCR analysis of ERdj3 in HeLa cells expressing R468Q IDS (*N* = 3). Cells were transfected with non-targeting siRNA or siRNA targeting ERdj3 (mean ± SD, *N* = 3, Student's *t*-test, ****P* < 0.001). The numbers indicate the percentages for control mRNA. **b** Quantification of the relative protein level of ERdj3 in (**Fig. 6a**) (mean ± SD, *N* = 3, Student's *t*-test, ****P* < 0.001). **c** Quantitative PCR analysis of ERdj3 in HeLa cells expressing A85T IDS (*N* = 3). Cells were transfected with non-targeting siRNA or siRNA targeting ERdj3 (mean ± SD, *N* = 3, Student's *t*-test, ****P* < 0.001). The numbers indicate the percentages for control mRNA. **d** Quantification of the relative protein level of ERdj3 in (**Fig. 6f**) (mean ± SD, *N* = 3, Student's *t*-test, ****P* < 0.001).

**Supplementary Fig. 6. Double knockdown of HRD1 and ERdj3, and shutdown of ERAD combined with a pharmacological chaperone do not augment recovery effects on the functions of IDS mutants.**

**a** Western blotting analysis of IDS in HeLa cells expressing R468Q IDS mutants (*N* = 3). Cells were transfected with non-targeting siRNA or siRNA targeting HRD1 and ERdj3. The knockdown cells were treated with 10 μM D2S0 for 48 h^46^. **b** Western blotting analysis of IDS in HeLa cells expressing A85T IDS mutants (*N* = 3). Cells were transfected with non-targeting siRNA or siRNA targeting HRD1 and ERdj3. The knockdown cells were treated with 0.1 μM D2S0 for 48 h^46^. **c** Quantification of the relative protein levels of mature forms of A85T IDS in (**b**) (mean ± SD, *N* = 3).

**Supplementary Table 1.**

Each specific primer set used to construct plasmids and RT-PCR.
